# Long-term sensory stimulation therapy improves hand function and restores cortical responsiveness in patients with chronic cerebral lesions. Three single case studies

**DOI:** 10.3389/fnhum.2012.00244

**Published:** 2012-08-25

**Authors:** Jan-Christoph Kattenstroth, Tobias Kalisch, Sören Peters, Martin Tegenthoff, Hubert R. Dinse

**Affiliations:** ^1^Neural Plasticity Laboratory, Institute for Neuroinformatics, Ruhr-University BochumBochum, Germany; ^2^Department of Neurology, BG-Kliniken Bergmannsheil, Ruhr-University BochumBochum, Germany; ^3^Department of Radiology, BG-Kliniken Bergmannsheil, Ruhr-University BochumBochum, Germany

**Keywords:** chronic cerebral lesion, repetitive sensory stimulation, sensorimotor hand function, single case

## Abstract

Rehabilitation of sensorimotor impairment resulting from cerebral lesion (CL) utilizes task specific training and massed practice to drive reorganization and sensorimotor improvement due to induction of neuroplasticity mechanisms. Loss of sensory abilities often complicates recovery, and thus the individual's ability to use the affected body part for functional tasks. Therefore, the development of additional and alternative approaches that supplement, enhance, or even replace conventional training procedures would be advantageous. Repetitive sensory stimulation protocols (rSS) have been shown to evoke sensorimotor improvements of the affected limb in patients with chronic stroke. However, the possible impact of long-term rSS on sensorimotor performance of patients with CL, where the incident dated back many years remains unclear. The particular advantage of rSS is its passive nature, which does not require active participation of the subjects. Therefore, rSS can be applied in parallel to other occupations, making the intervention easier to implement and more acceptable to the individual. Here we report the effects of applying rSS for 8, 36, and 76 weeks to the paretic hand of three long-term patients with different types of CL. Different behavioral tests were used to assess sensory and/or sensorimotor performance of the upper extremities prior, after, and during the intervention. In one patient, the impact of long-term rSS on restoration of cortical activation was investigated by recording somatosensory evoked potentials (SEP). After long-term rSS all three patients showed considerable improvements of their sensory and motor abilities. In addition, almost normal evoked potentials could be recorded after rSS in one patient. Our data show that long-term rSS applied to patients with chronic CL can improve tactile and sensorimotor functions, which, however, developed in some cases only after many weeks of stimulation, and continued to further improve on a time scale of months.

## Introduction

Sensorimotor impairment resulting from cerebral dysfunction has substantial physical, psychological and social implications. Generally, rehabilitation based on neuroplasticity mechanisms utilizes task specific training and massed practice to drive reorganization and improve sensorimotor function [for review see (Taub et al., 1999)]. However, somatosensory input is not only crucial for tactile and haptic but also for sensorimotor performance. Loss of sensory abilities, for example of the upper extremities, further complicates the possible recovery of motor functions and thus the individual's ability to use them for functional tasks. Therefore, the development of additional and alternative approaches that could supplement, enhance, or even replace conventional training procedures would be advantageous. In the last years, many attempts have been made to search for additional rehabilitative approaches [for review see (Johansson, 2011)].

Based on our studies employing repetitive stimulation protocols in healthy individuals, which demonstrated substantial improvements of tactile, haptic, and sensorimotor performance (Dinse et al., 2005, 2006, 2011; Kalisch et al., 2008a, 2010), we assumed that such approaches should evoke positive effects also in patients with cerebral lesions (CLs). In fact, studies in stroke patients revealed significant beneficial effects of a few weeks of repetitive stimulation (Smith et al., 2009).

The particular advantage of repetitive stimulation is its passive nature, which does not require active participation or even attention of the subjects. Therefore, repetitive stimulation can be applied in parallel to other occupations, making the intervention substantially easier to implement and more acceptable to the individual. We therefore initiated single case studies, were we treated individuals in which cerebrovascular dysfunctions dated back up to 13 years. The rational was to induce plastic processes within and around those brain areas that became dysfunctional. In all cases, repetitive stimulation was applied at the homes on a regular basis (5 days a week, for 45–60 min per day) using computer-controlled devices that monitored times and durations of stimulation sessions.

Here we report the effects of repetitive sensory stimulation (rSS) on the paretic hand of three patients treated over a time-period up to 76 weeks. To evaluate changes of sensorimotor functions elicited by rSS we assessed besides touch threshold and grip strength, motor performance like aiming, tapping, pin plugging, steadiness and multiple-choice reaction times. For the assessment of functional hand motor skills we used subtests from the Jebsen-Taylor Hand Function Test. Additionally, in one subject the possible impact of repetitive stimulation on brain organization was investigated by recording tactile-stimulation evoked somatosensory evoked potentials (SEP) using high-density EEG (Montoya and Sitges, 2006; Wienbruch et al., 2006). The purpose of the single case studies was to determine the effects of long-term rSS on the sensorimotor performance of patients with chronic CL who were considered resistant to any further standard therapy. Our data show that long-term rSS applied to the affected hand of patients with chronic CL can improve tactile and sensorimotor functions, which, however, developed in some cases only after many weeks of stimulation, and continued to further improve on a time scale of months.

## Materials and methods

### Subjects

At baseline before intervention, all three subjects had severe sensorimotor paresis as assessed using the Rivermead Motor Assessment arm section evaluating the functional motor capacity of the upper limb (Lincoln and Leadbitter, 1979; Woldag et al., 2003). Mini-Mental State Examination scores (Folstein et al., 1975) ranged from 28 to 30. All subjects gave their written informed consent. The local ethics committee of the Ruhr-University Bochum approved the protocol.

**Figure 1:**
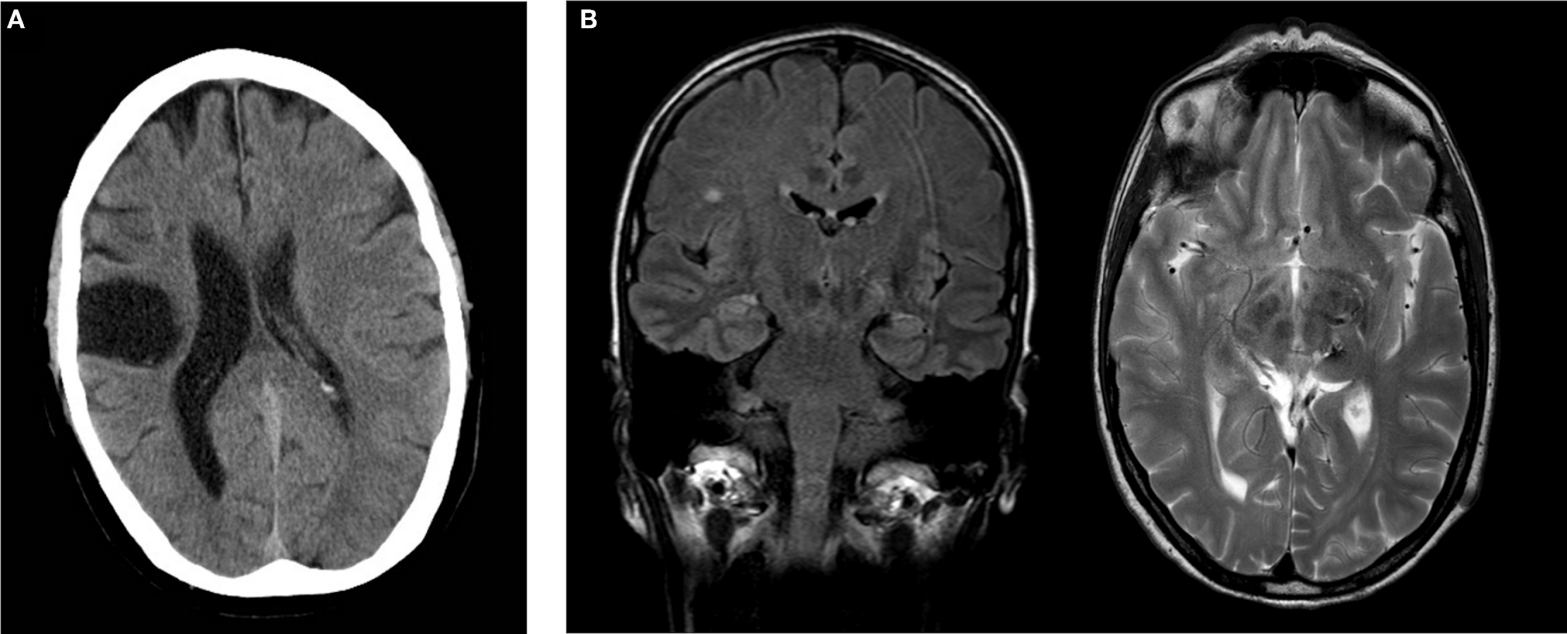
**(A)** Postischemic cranial CT of subject AA showing extensive postischemic necrosis in the pericentral right MCA distribution. **(B)** Postoperative cranial MRI of subject CC demonstrating a focus of gliosis in the left thalamus with discreet hemorrhagic residua on a T2w image. Incidental finding of single subcortical microangiopathic gliosis in the right centrum semiovale on FLAIR image. Coronal FLAIR image (left), transverse T2w image (right).

**AA**, male, born 1958, right-hemispheric ischemic CL in 2005. Baseline findings: discrete left brachiofacial sensorimotor hemiparesis with pronator drift and increased deep tendon reflexes, and left-sided hypaesthesia, particularly thermal hypaesthesia. AA agreed to report his medical history and actual medication. His Mini Mental Status Examination (MMSE) revealed 29 points. Postictal CT scans showed residua of a right-sided MCA infarct with areas of extensive cortical and subcortical colliquation necrosis in the distribution of the pericentral arteries (Figure 1A).

**BB**, male, born 1941, right-hemispheric cerebral contusion in 2004 due to a boxing induced traumatic brain injury. Baseline findings: discrete left-sided brachiofacial ensorimotor hemiparesis with increased deep tendon reflexes and upgoing plantar reflex. BB agreed to report his medical history and actual medication. His MMSE revealed 28 points. MRI imaging divulged scattered subcortical and cortical punctate foci of hypointense signal on susceptibility-sensitive images in the right fronto- and temporopolar lobes with discreet analogous contre-coup findings in the contralateral temporal lobe, and discreet linear foci of hypointense signal of the dura in the right–sided cranium as residua of subdural hematoma.

**CC**, female, born 1961, thrice preoperative intracerebral hemorrhage 1996 and 1997 secondary to thalamic vascular malformation with successful operative resection in 1997. Baseline findings: right-sided brachiofacial sensorimotor hemiparesis with pronator drift and increased deep tendon reflexes, hemihypaesthesia of the right hemibody, right-sided dysdiadochokinesis and astereognosia. CC agreed to report her medical history and actual medication. Her MMSE revealed 30 points. Postoperative MRI imaging disclosed a focus of postoperative gliosis centered on the left thalamus involving the ipsilateral cerebral crus, with discreet hemorrhagic residua on susceptibility-sensitive images (Figure 1B).

### Study design

We used a single-subject AB design for three independent subjects for both the affected and the non-affected hand/arm with baseline assessment before intervention, and multiple assessments during the intervention (Zhan and Ottenbacher, 2001). During intervention no additional standard therapy was administered. Data of the non-affected hand served as reference for estimation of intervention-induced effects.

### Repetitive sensory stimulation (rSS)

The rSS was applied for 45 min per day on the paretic hand of the patients at their homes after providing detailed instruction. The stimulation sequence was the same as described before (Ragert et al., 2008) and consists of 20 Hz bursts for 1 s with 5 s intertrain intervals and a ramp/fall time of 0.5 s and 0.2 ms pulse width [square]. The pulse trains were delivered with a two-channel stimulation device (ELPHA *II 3000*, danmeter, Danmark). To take into account the nervous innervation of the fingers, the stimulation of the predominantly N. medianus-innervated fingers d1–d3 (thumb, index- and middle finger) and the predominantly N. ulnaris-innervated fingers d4 and d5 (ring- and little finger) were separately controlled and delivered. The pulses were transmitted by adhesive surface electrodes (1 × 4 cm, Pierenkemper, Germany) fixed on the first and third segment of each finger (cathode proximal) [cf. (Dinse et al., 2008; Smith et al., 2009)]. The intensity of the stimulation was set individually at the highest threshold the patient could easily tolerate for the extended period of time resulting in an average stimulation intensity (N. medianus and N. ulnaris) of 14.8 ± 1.92 mA/9 ± 1 mA for subject AA, 64.56 ± 14.33 mA/42.37 ± 5.22 mA for subject BB and 25.25 ± 7.06 mA/18.71 ± 6.86 mA for subject CC.

### Cognitive performance

Based on figural reasoning, general intelligence was assessed once at baseline assessment using the Raven Standard Progressive Matrices (*RSPM*), a non-reading, non-language based measure of fluid intelligence (Raven, 1938).

### Everyday competence

Lifestyle and general activity level was assessed once at baseline assessment using the “Everyday Competence Questionnaire” (*ECQ*) addressing aspects of everyday life like independence in activities of daily living and mobility, social relations, general health status, and life contentment (Kalisch et al., 2011b).

### Multiple-choice reaction time measurement (RT)

We performed multiple-choice RT measurements in a finger-selection visuo-tactile task adapted from the study of (Alegria and Bertelson, 1970; Wilimzig et al., 2012). Subjects were seated 3 m in front of a monitor. An image of each hand was displayed on the monitor and one finger of the 10 was selected by a visual marker. Subjects had to press the key corresponding to the selected finger on a hand-shaped, 10-button keyboard as fast as possible. One session consisted of four blocks of 100 trials each, which were separated by a short break after each block. The maximum response-to-stimulus interval for each trial was 2000 ms. Each finger was tested 40 times in a random order.

### Quantitative handedness assessment

Handedness was assessed in subject CC using the “hand-dominance test” (HDT) (Steingrueber, 1971; Jäncke et al., 1997, 2000), which comprises three dexterity tasks, each to be performed with maximal speed and precision over 30 s, separately for the right and left hand (line tracing, dotting circles, and dotting squares). Performance was scored for each hand and the percentile rank (PR) was calculated according to the classification provided by the HDT manual. Classifications were PR < 3 = extreme left-handedness, PR 3–8 = left-handedness, PR 9–16 = ambidexterity, PR 17–79 = right-handedness, and PR > 79 = extreme right-handedness.

### Motor performance

Hand-arm fine-motor performance was evaluated using a commercial, computer-based test-battery for clinical neuropsychological research (MLS, Dr. G. Schuhfried GmbH, Mödling, Austria) as described previously (Kalisch et al., 2006). The system consists of a work plate with two pencils for left and right hand use. All parts of the system are connected to an interface and a PC computer to record the time and number of errors during different tasks. We measured speed, accuracy, and maintenance of upper limb position during execution of fine motor movements of the affected and non-affected arm, hand, and fingers using following tests.

### Steadiness

Steadiness evaluates the ability to obtain a prescribed arm-hand position and to maintain it for a defined time period. Subjects were asked to place the pencil into a small circular hole (5.8 mm) of the horizontally positioned board, and hold it there without touching the edges for 32 s without support of the hand. This task tests the ability to hold a steady position, and allows an estimate of postural tremor. Dependent variables were the number of errors, i.e., the number of contacts of the pencil with the wall of the hole. Differences between affected and non-affected hand was evaluated using the steadiness ratio error duration / error.

### Aiming

Aiming evaluates the ability to accomplish fast arm-hand movements for small targets. Subjects had to consecutively hit 20 linearly arranged small contact fields (diameter 5 mm, midpoint separation 9 mm) with a test pencil. This test assesses the degree of ataxia and the speed of movement by the ability to make rapid repeated aimed movements. The dependent variables were the number of errors (missed contact fields) and the total time needed to complete the task.

### Pin plugging

Pin plugging evaluates fine and gross motor dexterity and coordination. The board carries two rows of 25 small holes, one on the left side and one on the right side. Two containers, each equipped with 25 metal pins, were placed in 30 cm distance from the right and left side of the board. Subjects were asked to pick the pins with her affected hand, one by one, from the right container and insert them into the holes on the peg-board. Subsequently the test was continued using the non-affected hand and left container. Time to complete the test was assessed. The test was performed in a standard version (size of metal pins 5 × 0.25 cm) and in a more demanding version with smaller pins (size of metal pins 1 × 0.25 cm).

### Tapping

Tapping evaluates the ability to perform very fast, repetitive wrist-finger movements with little emphasis on precision of movement. Subjects were required to hit a square contact plate (40 by 40 mm) on the test board with a test pencil as frequently as possible. The measured parameter was number of hits achieved in a time interval of 32 s, which provides a measure of the speed of antagonistic oscillation. In this task, support of the forearm was allowed. Therefore, the repetitive contacts had to be accomplished by wrist movements.

### Grip strength

Grip strength was measured three times for each hand with a Jamar hand dynamometer (Sammons Preston Inc., Bolingbrook, IL). Subjects were asked to stand up and hold the dynamometer with the arm parallel to the body.

### Tactile performance

#### Touch threshold

Touch thresholds were evaluated by probing the fingertips with von Frey filaments (Marstocknervtest, Marburg, Germany). Each filament was calibrated to a known buckling force determined by its length and diameter. The test kit contained 16 different filaments calibrated to forces ranging from 0.08 to 294 mN in logarithmic scaling. Fine-touch sensitivity was tested with a staircase procedure, during which the subject was required to indicate whenever an indentation was perceived. The applied contact forces were decreased in a stepwise manner until the subject no longer perceived the stimulus (lower boundary), and then increased until the stimulus was perceived again (upper boundary). This procedure was repeated three times, resulting in six values that were averaged to form the absolute touch threshold. Differences between affected and non-affected hand was evaluated in percent difference.

#### Two-point discrimination threshold

Spatial 2-point discrimination thresholds (*2pd*) were assessed on the tips of the left (LID) and right (RID) index fingers by using the method of constant stimuli (Dinse, 2006; Kalisch et al., 2007, 2008b). Needle separations of 1.5, 2.3, 3.1, 3.9, 4.7, 5.6, and 7 mm were used. Test-retest reliability using this procedure was 0.90 for young subjects and 0.88 for older participants (Dinse et al., 2006). The summed responses were plotted against the needle distances resulting in a psychometric function, which was fitted using a binary logistic regression (SPSS, IBM, USA). The threshold was taken from the fit where 50% correct responses were reached.

### Jebsen-taylor hand function test (JTHFT)

For the assessment of functional hand motor skills we used subtests from the Jebsen-Taylor Hand Function Test (**JTHFT**) (Jebsen et al., 1969; Hackel et al., 1992). Six of the seven JTHF subtests had to be performed. (1) Moving heavy cans (**HC**) (250 g, diameter: 7 cm, 10.5 cm height), (2) Moving light cans (**LC**) (50 g, diameter: 7 cm, 10.5 cm height), (3) Picking up small objects placing them in a can (**SOP**), (4) Picking up small objects with a teaspoon placing them in a can (**FEED**), (5) Stacking checkers (**STACK**), (6) Turning cards (**TURN**). For analysis each subtest was videotaped for offline-analysis with VIANA 2.64 (University Essen, Germany).

### Recording of somatosensory evoked potentials

SEP were recorded after pneumatic stimulation of the left and right index (d2) and little finger (d5) prior and after intervention using high density EEG. We here report results of SEP recordings for subject CC only, as recordings of subject BB were not reliably interpretable due to mechanical deformations after traumatic injury and following surgery. We choose pneumatic stimulation as it has been shown that this form of fingertip stimulation activates the human primary sensory cortex (Elbert et al., 1995; Candia et al., 2003), while electrical stimulation activates afferent fibers (Hashimoto et al., 2004). Stimulation was conducted as described by Wienbruch and colleagues (2006). Non-painful stimuli of 50 ms duration, with a frequency of 1.5 Hz were applied to finger clips with flexible membranes placed on each finger segment of d2 and d5. The stimulus device (Department of Psychology, University of Konstanz, Germany) generates TTL pulses using incoming signals from a programmable pulse generator (AMPI, Jerusalem, Israel). TTL pulses were used to drive a magnetic solenoid valve (525146 MHE3-M1H-3/2G-1/8; Festo®, Germany; operating pressure: 0.9–8 bar). The incoming airflow drives a circular synthetic membrane of approximately 0.8 cm^2^ (4D Neuroimaging Inc., San Diego, USA) through plastic standard tubes (PUN-4×0,75-SI; outside diameter 4 mm, inside diameter 2.6 mm and PUN-3×0,5-SI; outside diameter 3 mm, inside diameter 2.1 mm; copper and Teflon-free for QS push-in fittings, Festo®) [cf. (Wienbruch et al., 2006)]. During measurements subjects were instructed to concentrate on the stimulus. SEP were obtained from 256 electrodes referenced to Cz (vertex electrode) using Geodesic Sensor Net and Net amplifiers (Electrical Geodesics Inc., Eugene, OR). Electrode impedance was below 50 kΩ. All signals were sampled at a rate of 1000 Hz with a high-pass filter of 0.1 Hz. The continuous data stream was cut into epochs. Before averaging, all 256 channels were scanned for artifacts, defined as amplitudes (max – min) larger than 200 μV. Further analysis was done using Brain Electrical Source Analysis (BESA, MEGIS Software GmbH, Munich, Germany). For waveform analysis all channels were band-pass filtered (0.4 Hz – 40 Hz (24 db/oct)) and digitally converted to international 10–10 system. In accordance with Montoya and coworkers, waveform analysis were conducted considering the tactile evoked potentials consisting of P50, N80 and P200 (Montoya and Sitges, 2006).

### Video-based analyses of manual dexterity in patient BB

We investigated manual dexterity of the affected hand to evaluate the ability to perform exploratory hand/finger movements for the haptic exploration of three-dimensional objects (Lederman and Klatzky, 2004; Kalisch et al., 2011a). For this purpose the subject was asked to manually explore a simple geometric object without viewing them, and to report sensations. The objects used were shown to the subject before the test started.

## Results

### Subject AA

**Figure 2:**
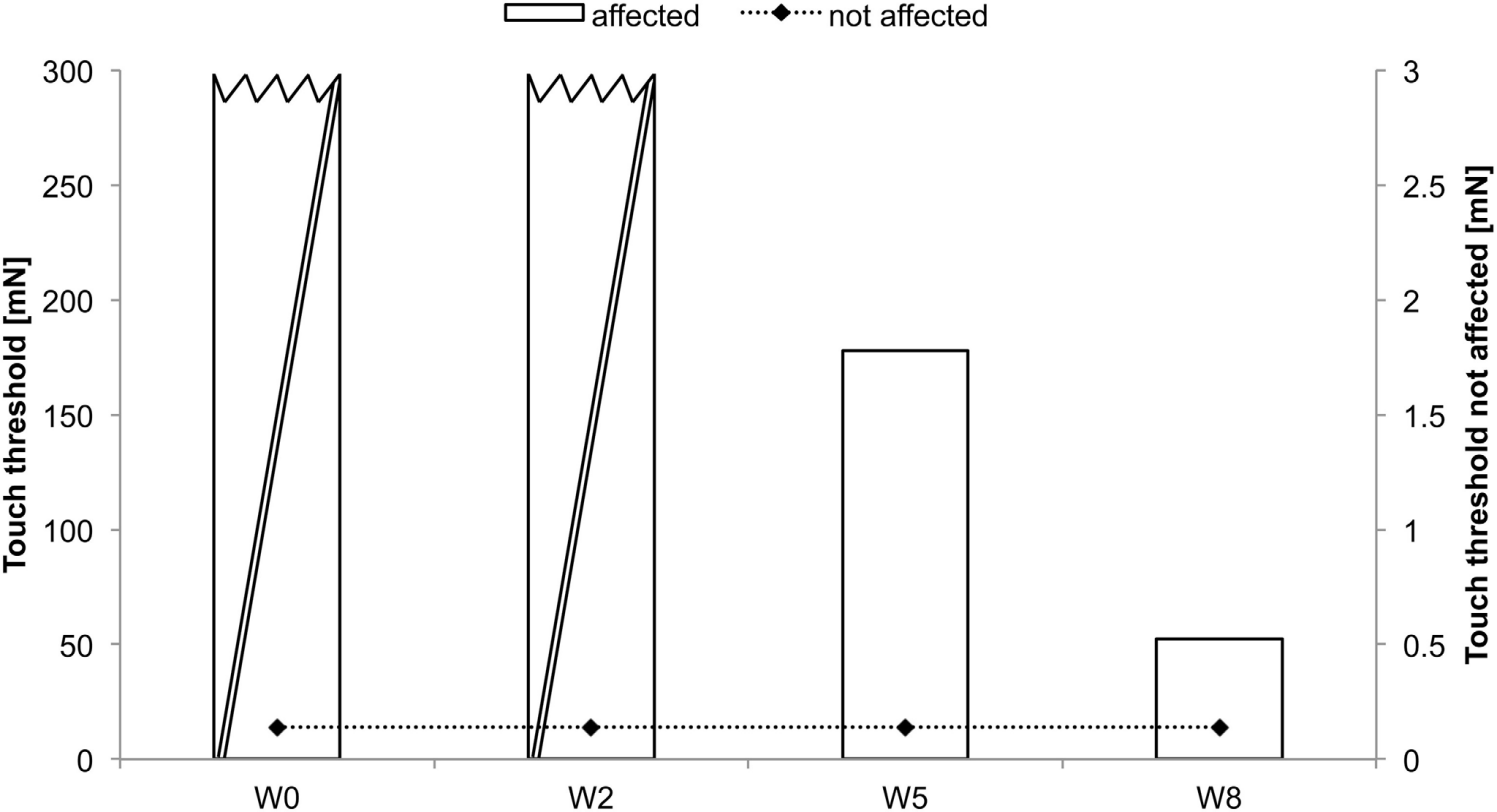
**Absolute touch thresholds of the index fingers (IF) for subject AA**. While no sensation was reported prior and after 2 weeks of intervention at the paretic limb using forces of 294 mN (crossed out bars), touch thresholds were substantially reduced and thus measurable after 5 (117.78 mN) and 8 (52.62 mN) weeks of intervention. Touch thresholds of the unaffected limb were only assessed prior to the intervention.

AA received rSS for a total of 8 weeks. For an overview of metadata and performance assessed at various time points see Table 1. Assessments were performed at baseline, and after 2, 5, and 8 weeks of intervention for touch threshold and 2-point discrimination. After 8 weeks of rSS we found substantial improvements in tactile performance (Table 1). Figure 2 shows the time course of improvement of touch thresholds during the 8 weeks of intervention. While at the paretic limb no sensation was measurable prior and after 2 weeks of intervention, touch thresholds could be assessed after 5 (117.78 mN) and 8 (52.62 mN) weeks of intervention. Comparable improvements were found for 2-point discrimination thresholds, which revealed a similar time course during intervention.

**Table 1:** **Cognitive, motor, tactile and activity performance of subject AA**.

**Variables**	**W0**		**W2**		**W5**		**W8**	
	**not affected**	**affected**	**not affected**	**affected**	**not affected**	**affected**	**not affected**	**affected**
Age [years]	48							
Everyday competence (ECQ)								
Rivermead motor assessment (RMA)	8							
Mini Mental Status Examination (MMSE)	30							
**TACTILE PERFORMANCE**								
Touch threshold [mN], *d1*	0.15	243.50		181.50		78.40		83.55
Touch threshold [mN], *IF*	0.14	255		255		117.78		52.62
Touch threshold [mN], *d3*	0.21	232		166.67		28.37		30.12
Touch threshold [mN], *d4*	0.17	255		219		44.45		31.28
Touch threshold [mN], *d4*	0.22	255		243.50		35.47		31.28
2-Point discrimination threshold [mm], *d1*	1.87	7		7		6.04		5.61
2-Point discrimination threshold [mm], *IF*	3.18	7		7		6.35		5.40
2-Point discrimination threshold [mm], *d3*	3.50	7		7		6.96		5.87
2-Point discrimination threshold [mm], *d4*	4.99	7		7		6.04		5.29
2-Point discrimination threshold [mm], *d5*	3.99	7		7		6.00		5.12

IF, index finger.

### Subject BB

**Figure 3:**
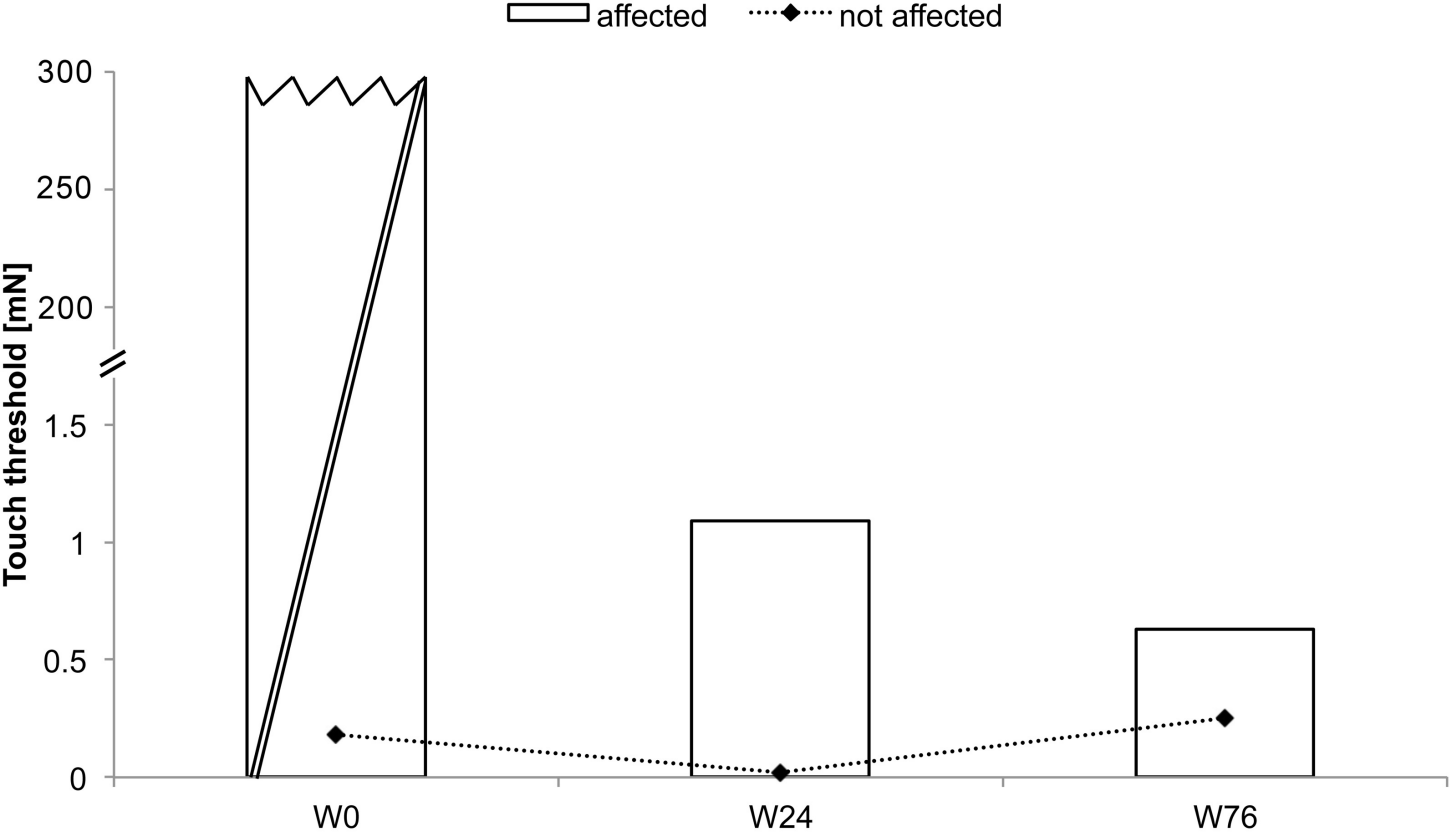
**Absolute touch thresholds of the index fingers (IF) for subject BB (note split ordinate)**. While no assessment was possible prior to the intervention using maximal forces of 294 mN (crossed out bar), touch thresholds were markedly reduced to 1.09 mN after 24 weeks and to 0.63 mN after 76 weeks of intervention.

**Figure 4:**
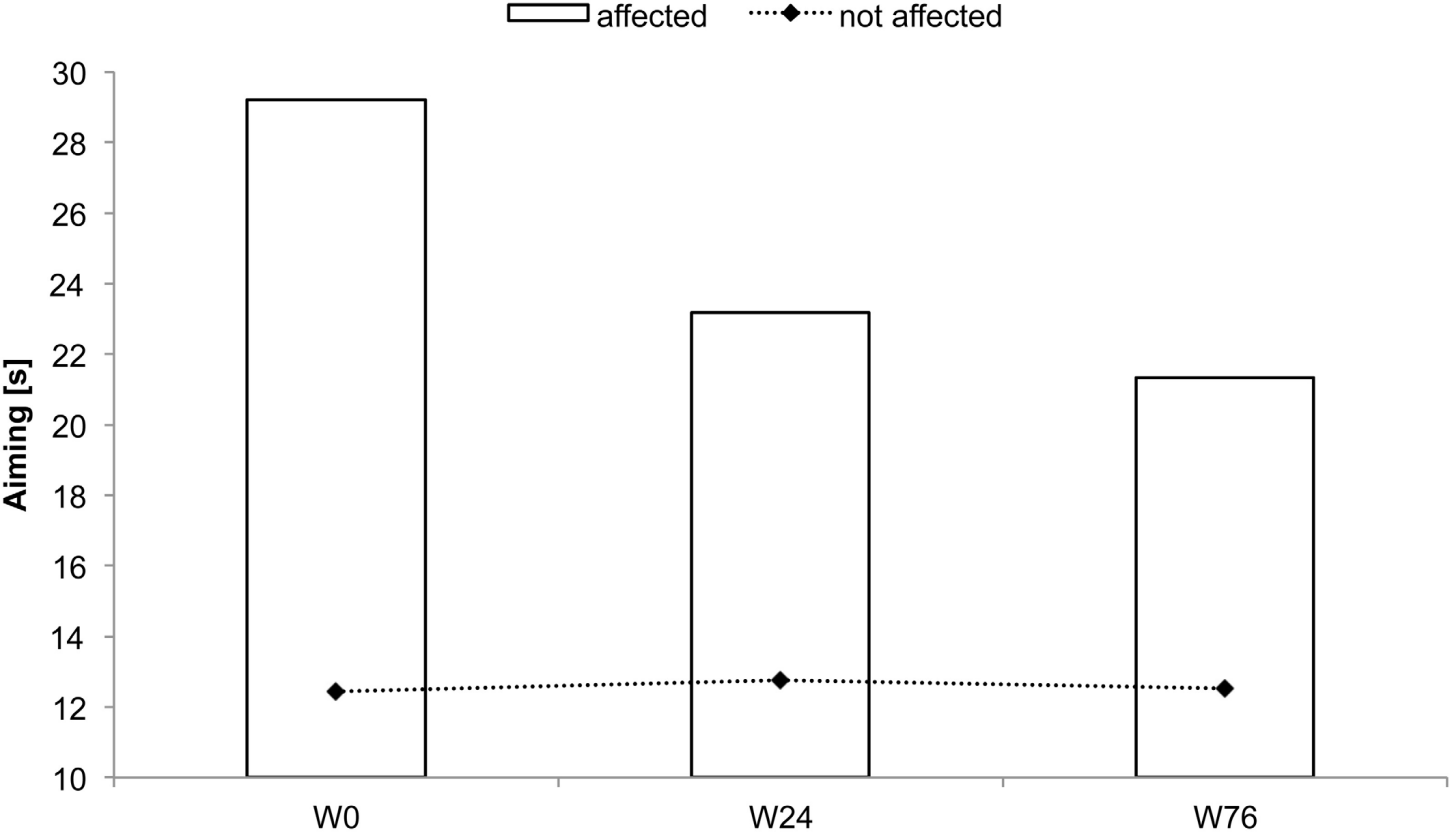
**Time to complete the aiming test for subject BB**. Total time decreased after 24 and 76 weeks of intervention.

BB received rSS for a total 76 weeks. For an overview of metadata and performance assessed at various time points see Table 2. At the affected hand the assessment of absolute touch-threshold was possible after 24 weeks of rSS (Figure 3), while 2-point discrimination thresholds could be assessed after 76 weeks of rSS. As shown in Table 2 motor performance improved for the time needed for the execution of the aiming task (Figure 4), while the number of errors remained constant. Using the affected hand, execution of the demanding version of the pin-plugging task with short pins was possible after 76 weeks of rSS intervention. No improvements were found for the standard pin-plugging task (long pins) and for maximum tapping rates. Grip strength increased gradually over the intervention period both for the unaffected and the affected hand.

**Table 2:** **Cognitive, motor, tactile and activity performance of subject BB**.

**Variables**	**W0**		**W24**		**W76**	
	**not affected**	**affected**	**not affected**	**affected**	**not affected**	**affected**
Age [years]	69					
Everyday competence (ECQ)	27					
Rivermead motor assessment (RMA)	5					
Mini mental status examination (MMSE)	28					
Raven standard progressive matrices (RSPM)	40					
**MOTOR PERFORMANCE**						
***Control Precision***						
Aiming [error]	0	0	0	0	0	1
Aiming [s]	12.43	29.2	12.75	23.18	12.53	21.34
Pin plugging [s], short	89.87	*n.p.*	74.34	*n.p.*	73.43	166.48
Pin plugging [s], long	63.81	127.61	62.79	115.86	66.07	141.58
***Rate of wrist movement***						
Tapping [hits]	165	110	167	125	158	120
**TACTILE PERFORMANCE**						
Touch-threshold [mN], *IF*	0.18	*n.p*.	0.08	1.09	0.25	0.63
2-Point discrimination threshold [mm], *IF*	3.5	*n.p*.	3.61	*n.p*.	3.6	4.84
**Grip strength [kg]**	28.3	26.6	41.2	28	48	35

IF, index finger; n.p, Assessment not possible.

### Video-based analyses of manual dexterity in patient BB

#### Video 1 (see supplementary information online)

The pre-video recording was performed during the baseline assessment (week 0) with subject BB. Apparently, BB was not able to manipulate the objects, and as a result, was not able to recognize the objects. This failure was due to his inability to feel the position of the object in his hand and to control the object position though manipulative movements. Because of these limitations, BB reported that he was not sure if the object was still in his hand or not.

#### Video 2 (see supplementary information online)

The second post-video recording was carried out after 76 weeks of daily application of electrical repetitive stimulation (rES). The manipulatory abilities of the affected hand were substantially improved. BB was able to grab the object with all fingers of the hand and to perform controlled exploratory movements with the thumb, index and middle fingers. Furthermore, BB was now able to use the thumb for a more detailed exploration of surface curvatures. As a result, BB was able to identify almost every object after a short exploration phase.

### Subject CC

**Figure 5:**
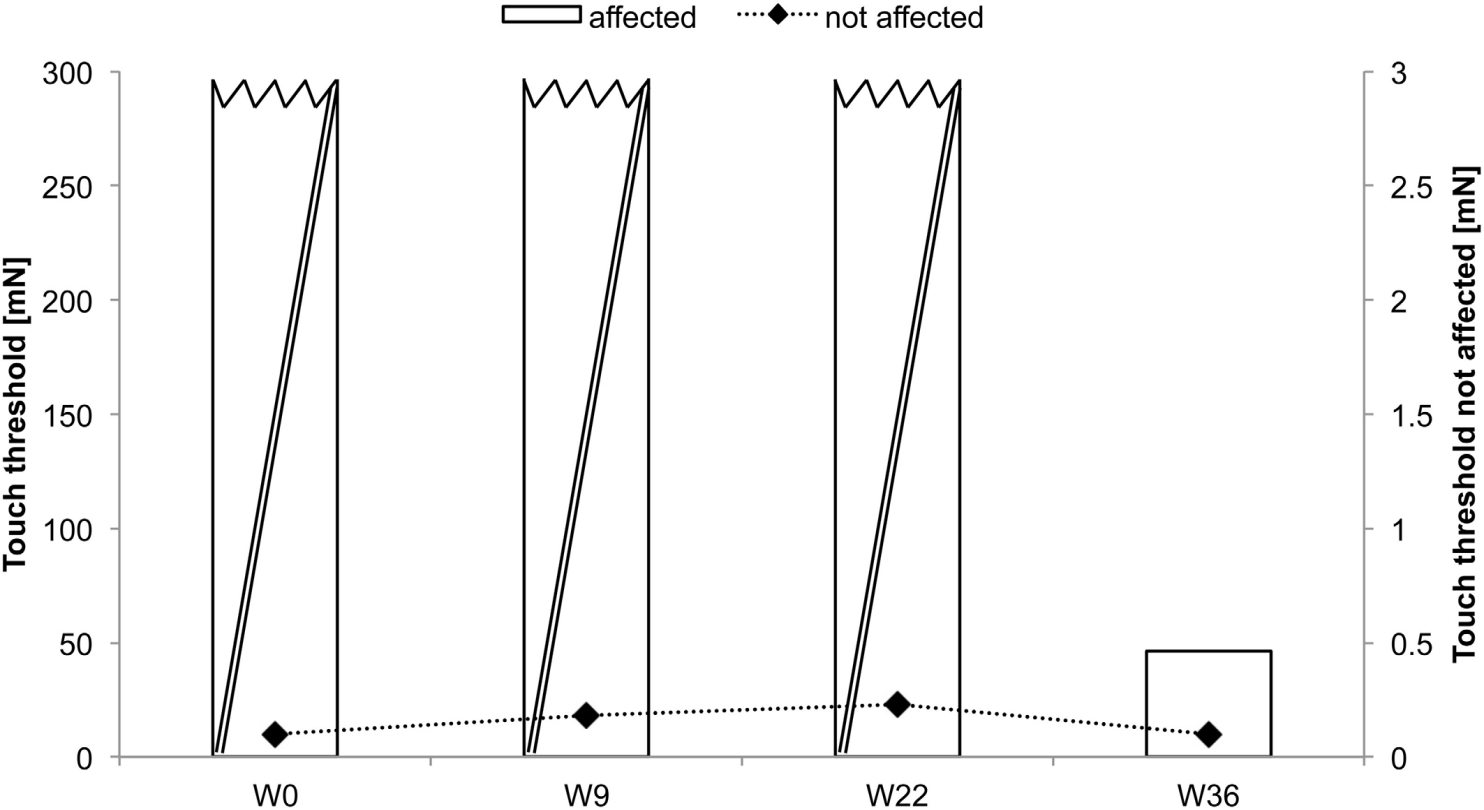
**Absolute touch thresholds of the index fingers (IF) for subject CC**. While no assessment was possible prior and after 9 and 22 weeks of intervention at the paretic limb using forces of 294 mN (crossed out bars), touch thresholds were reduced and thus measurable after 36 weeks (46.28 mN) of intervention.

**Figure 6:**
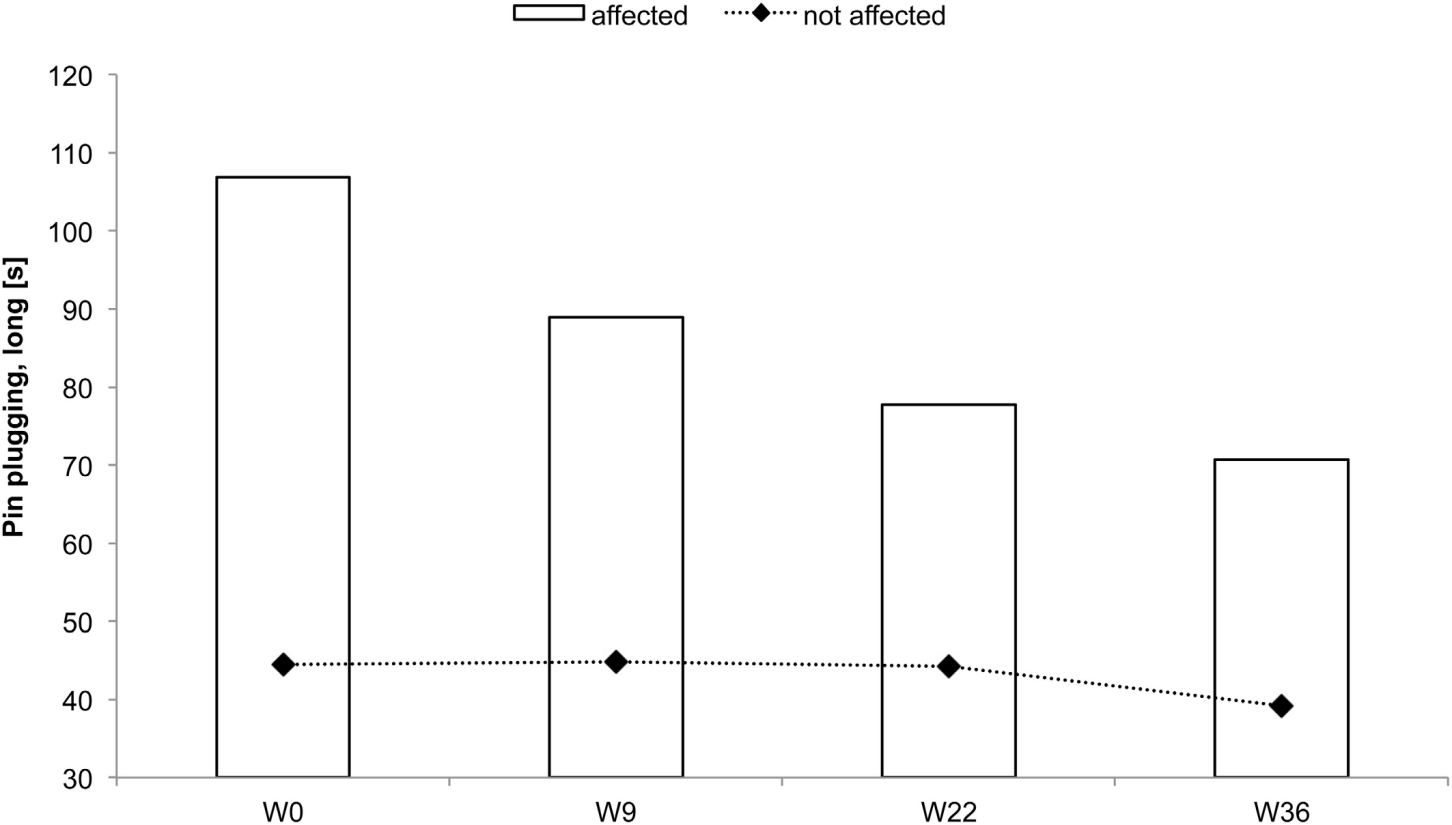
**Total time to complete the pin-plugging test using long pins for subject CC**. Improvements were found after 9 weeks of intervention for the affected hand.

**Figure 7:**
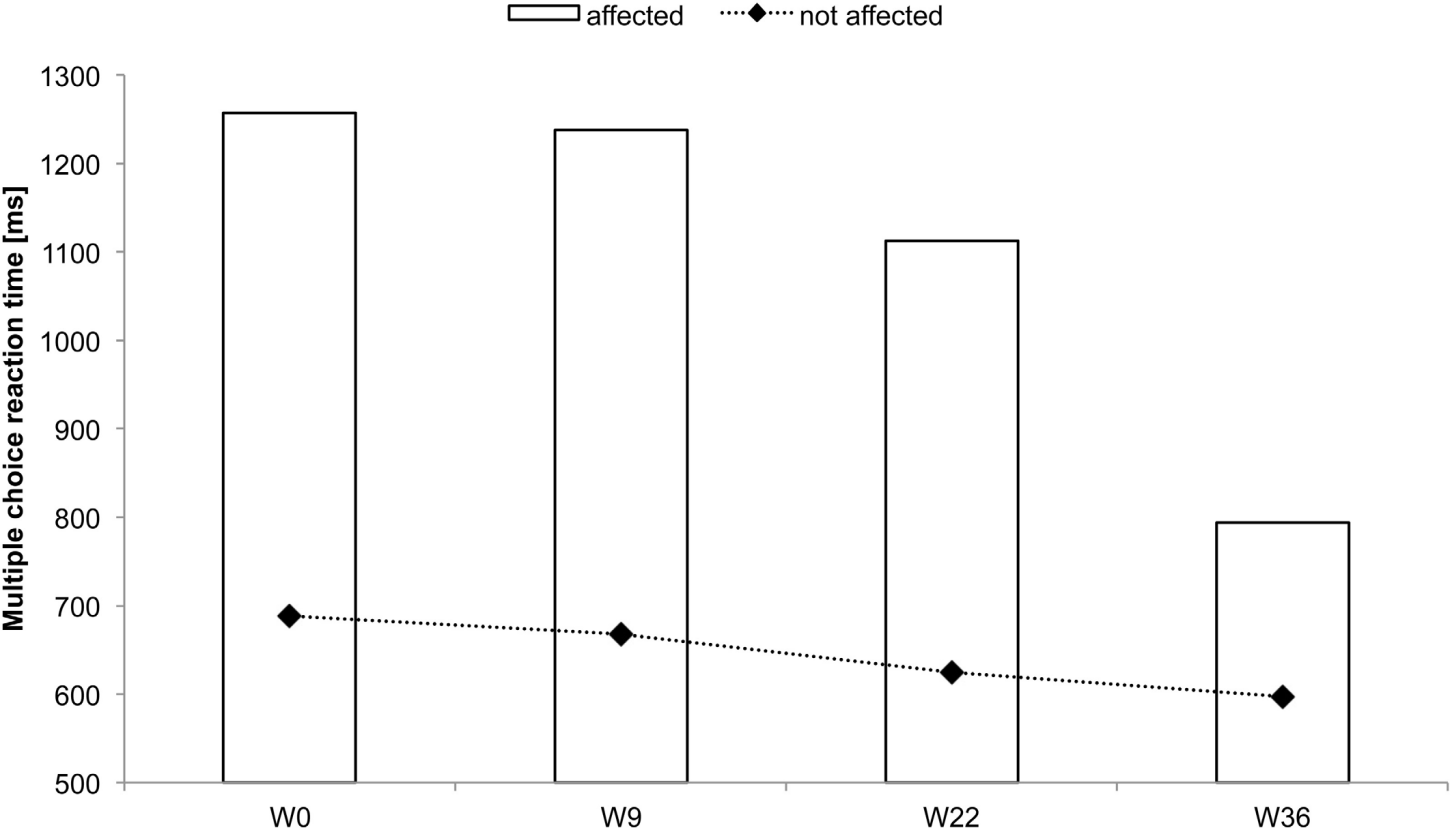
**Multiple-choice reaction times of all fingers averaged for each hand for subject CC**. Considerable reaction time speeding up was found for the affected hand after 22 and 36 weeks of intervention.

**Figure 8:**
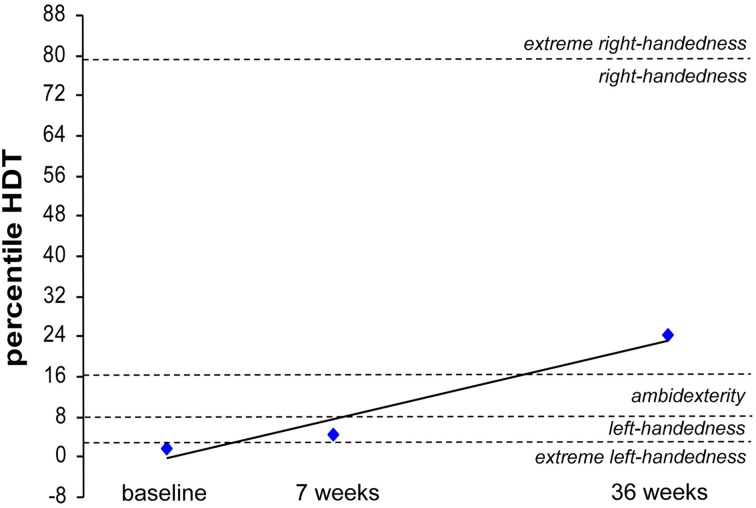
**Percentile rank of the hand-dominance-test (HDT) for subject CC at baseline, after 7 weeks and after 36 weeks of intervention**. Extreme left-handedness (PR = 1.7) was found at baseline, changing to left-handedness (PR = 4.2) after 7 weeks of rSS intervention resulting in right-handedness (PR = 24) after 36 weeks of intervention.

CC received rSS for a total 36 weeks. For an overview of metadata and performance assessed at various time points see Table 3. Touch thresholds at the affected hand could not be assessed until the fourth assessment after 36 weeks of intervention (Figure 5), while motor performance assessment revealed considerable improvements for subject CC after 36 weeks of rSS. Pin-plugging using long pins showed improvements after 9 weeks of intervention (Figure 6), while multiple-choice reaction times considerably improved after 22 weeks of rSS (Figure 7). Prior to the incident, CC was right-handed. At baseline the handedness assessment revealed an extreme left-handedness (PR = 1.7), but gradually changed from left-handedness (PR = 4.2) after 7 weeks of rSS intervention to right-handedness (PR = 24) after 36 weeks of intervention (Figure 8).

**Table 3:** **Cognitive, motor, tactile and activity performance of subject CC**.

**Variables**	**W0**		**W9**		**W22**		**W36**	
	**not affected**	**affected**	**not affected**	**affected**	**not affected**	**affected**	**not affected**	**affected**
Age [years]	49							
Everyday competence (ECQ)	28							
Rivermead motor assessment (RMA)	6							
Mini mental status examination (MMSE)	30							
Raven standard progressive matrices (RSPM)	49							
**REACTION TIMES**								
Multiple choice reaction times [ms]	688.25	1257.02	667.92	1238.1	624.68	1112.5	597.32	794.16
**MOTOR PERFORMANCE**								
***Control Precision***								
Aiming [error]	1	2	1	6	1	13	2	5
Aiming [s]	10.11	20.45	8.62	11.59	9.54	12.43	9.47	10.67
Pin plugging [s], short	53.93	130.45	52.89	102.62	43.99	101.48	57.64	127.38
Pin plugging [s], long	44.5	106.89	44.78	88.99	44.3	77.76	39.2	70.75
HDT [percentile rank]	1.7		4.2		n.a.		24	
***Rate of wrist movement***																
Tapping [hits]	178	150	170	145	172	161	184	160
***JTHFT [s]***								
Heavy cans					9.16	10.44	7.8	9
Light cans					7.44	10.4	7.04	9.56
Small object picking					9.52	19.92	9.96	17.04
Feeding					13.32	16.12	11.76	13.04
Stacking					3.28	4.8	3.04	6.4
Turning					6.76	15.12	5.84	6.4
**TACTILE PERFORMANCE**								
Touch-threshold [mN], *IF*	0.1	*n.p*.	0.18	*n.p*.	0.23	*n.p*.	0.1	46.28
**Grip strength [kg]**	19	16	19	14	20	16	18	19.3

IF, index finger, n.p, Assessment not possible.

**Figure 9:**
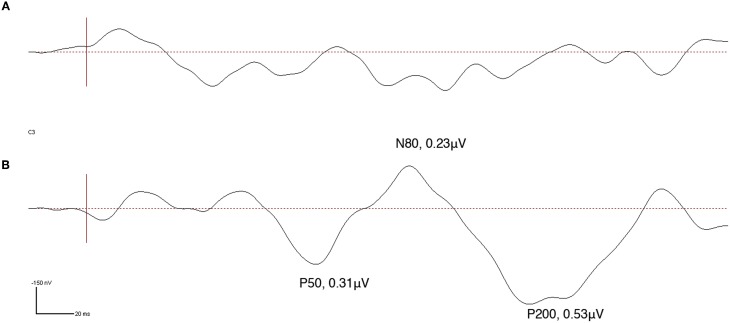
**Somatosensory evoked potentials (SEPs) obtained from high-density EEG recording at electrode C3 made in subject CC during tactile stimulation of the little finger (d5) of the affected hand**. SEP source waveforms were analyzed using brain electrical source analysis (BESA), MEGIS Software GmbH, Munich, Germany. Vertical red line represents stimulus onset. While prior to intervention no SEP was detectable **(A)**, a clear P50, N80, and P200 indicative of normalized tactile somatosensory cortex processing could be obtained after 22 weeks of intervention **(B)**.

Prior to intervention ipsi-lesional cortical SEPs were absent at the N. ulnaris innervated finger d5 (little finger) while after 22 weeks of intervention cortical SEPs could be recorded over C3. No SEPs could be recorded during the entire observation period for the N. medianus innervated finger d2 (index finger) (Figure 9). The clear emergences of the typical cortical SEP components after tactile stimulation implicate a partial restoration of processing of tactile information in SI.

## Discussion

We investigated the feasibility of long-term sensory stimulation-based (repetitive electrical stimulation of all fingers of the affected hand – rSS) therapy in three single patients characterized by cortical lesions affecting the upper extremities. In all three patients, at the time the intervention started, the incident dated back several years, between 2 and 11 years. The duration of intervention was 8, 35 and 76 weeks. For treatment, participating patients were equipped with stimulation devices which allowed daily stimulation sessions of 45 min per day on average at the homes. The detailed assessment of sensorimotor performance was different across participants, as a rule, we measured sensory performance (touch threshold and spatial acuity) and various aspects of motor abilities. Apparently, our study is not intended to imply any similarity or comparability between the three cases. In fact, each patient represents a highly individual case. Moreover, we used different batteries of behavioral tests in each subject, and electrophysiological testing has been conducted in only one of the patients. Despite this heterogeneity, our study aimed at demonstrating the broad applicability of our approach, which is not limited to narrowly defined forms of cortical injuries or lesions. Our data showed that rSS improved tactile and sensorimotor functions. However, beneficial effects developed only after weeks of stimulation, but continued to develop during maintained regular schedule of stimulation on a time scale of months. Of course, the possibility remains that placebo-like effects, or spontaneous healing processes might have been involved in mediating the overall beneficial effects. Therefore, further studies are needed to clarify the effectiveness of long-term treatment in patients with chronic CLs.

Over the last years, many different forms of peripheral nerve stimulation, mostly of the hand and fingers, have been studied in healthy and impaired human populations. Peripheral nerve stimulation has been shown to elicit activations in primary somatosensory cortex as measured by blood oxygenation level-dependent (BOLD) signal using fMRI (Pleger et al., 2003). At this, PNS increases motor cortical excitability beyond the period of stimulation (Hamdy et al., 1998; Ridding et al., 2000; Fraser et al., 2002; Kaelin-Lang et al., 2002; McDonnell and Ridding, 2006).

Most studies investigating the effects of peripheral nerve stimulation in stroke patients have reported improvement of functional hand tasks after a single application (Hamdy et al., 1998; Conforto et al., 2002; Kaelin-Lang et al., 2002; Ridding and Uy, 2003; McDonnell and Ridding, 2006; Wu et al., 2006; Conforto et al., 2007). Accordingly, behavioral improvements have been documented for assessments performed after the intervention, but few data are available about the long-term stability of the effects. Other studies investigated effects of combining peripheral nerve stimulation and motor training for rehabilitation (Wu et al., 2006; Celnik et al., 2007). Conforto and co-workers showed that median electric nerve stimulation could enhance performance of functional hand tasks even 30 days after training in a group of cortical stroke patients (Conforto et al., 2007). Comparable maintenance of positive effects were reported for chronic stroke patients in a follow up of 4 weeks (Smith et al., 2009). However, the present study represents a major extension of the Smith et al. study, where the maximal duration of treatment was 6 weeks, while we here treated patients up to 10 times longer. Furthermore, the Smith et al. study was restricted to stroke patients, while we here included other types of brain lesion/injuries in order to show the broad feasibility of a long-term stimulation approach.

Our data presented here demonstrate for the first time that this approach can also be efficiently used over many weeks in long-term patients with CLs independent of the individual etiology of the lesion, where the incident dates back many years. This is of particular importance, as alternative forms of treatment reveal practical limitations under constraints that require regular schedules of applications over a year or more.

All three patients were characterized by severe sensory deficits. For example, touch thresholds were not assessable using forces up to 294 mN. Similarly, spatial discrimination was significantly impaired. Nevertheless, during and after rSS, we observed substantial improvement of tactile abilities. It has been well recognized that the typical approach in neurorehabilitation after CLs consists of motor training (Nudo et al., 1996; Taub et al., 2002; Dobkin, 2004). Despite substantial advances in the development of effective training protocols, functional recovery is usually incomplete and most patients experience long-term disabilities (Celnik et al., 2007). In particular, beneficial effects on pure sensory functions are mostly limited following motor rehabilitation. Conceivably, largely intact somatosensory input is required for learning and re-learning of skillful motor behavior (Pavlides et al., 1993; Ebied, 2003; Celnik et al., 2007). When inputs are corrupted, motor behavior is corrupted as well (Celnik et al., 2007). It is therefore not surprising that patients with somatosensory deficits following stroke suffer more persistent motor impairment than those without such deficits (Celnik et al., 2007).

Over the last years, may attempts have been made to understand mechanisms mediating the recovery after cerebral damage, and much progress has been made through a combination of either animal models or the use of new stimulation protocols such as transcranial magnetic stimulation (Webster et al., 2006). Generally, there is agreement that many of the mechanisms that underlie recovery are similar to those mediating plasticity in the intact brain. Given that a damaging event spares some cortical or subcortical circuitry, which process sensory and motor information, synapse-based learning is the main substrate that can create compensatory circuits after brain damage (Murphy and Corbett, 2009). Processes that are crucial for recovery include the unmasking of latent subthreshold inputs and the formation of new synapses.

Experimental data from stroke animal models indicate that stroke causes an initial loss of cortical responsiveness to the impaired limb that is gradually restored and remerges predominately within the peri-infarct motor and hindlimb regions, the development of prolonged cortical sensory responses in local and distant regions, large-scale changes in neuronal connectivity with the retrosplenial cortex, primary and secondary somatosensory cortical regions, and the striatum, and finally enhanced turnover of the presumptive postsynaptic targets of cortical connections in the reorganized forelimb area such as dendritic spines for several weeks after stroke (Brown et al., 2009).

Moreover, following stroke an imbalance of interhemispheric excitability has been described, which is probably due to the reduced inhibition from the affected hemisphere, either mediated through transcallosal fibres, or through connections below the cortical level (Gerloff et al., 1998; Liepert et al., 2000, 2001). This opens the possibility of targeted intervention on the healthy ipsilateral hemisphere to induce beneficial effects on the affected side (Chen et al., 1997). Animal studies have suggested that the recruitment of the undamaged hemisphere may depend on the functional integrity of the remaining sensorimotor system (Biernaskie et al., 2005).

Combined, many different processes contribute to recovery. The heavy schedule of rSS we applied over prolonged periods of time is thought to induce plasticity processes. Because of the timing properties of the stimulation protocols, which consist of high-frequency intermittent stimulation, it has been assumed that the stimulation results in long-term potentiation-like effects, which facilitate reactivation of cortical tissue that has preserved some functionality. The resulting remodeling of cortical circuits is then assumed to mediate behavioral recovery (Dinse et al., 2011).

It is a remarkable observation that despite major impairments in sensation in each of the participants it was possible to record sensory thresholds for minimal sensations evoked by electrically stimulating the fingers. In particular, both patients who were not able to perceive a sensation of touch at maximal forces (294 mN), we could drive sensations using electrical stimulation though at much higher stimulation intensities than typically observed in healthy individuals. Apparently, it is possible by electrically stimulating nerve fibers to overcome the loss of cutaneous sensations mediated via mechanoreceptors.

Besides improvement of sensory abilities, long-term application of rSS also improved motor performance in both patients who underwent motor performance assessment. The efficacy of rSS to improve motor function had also been demonstrated for healthy adult and elderly individuals (Kalisch et al., 2007, 2008a, 2010; Kowalewski et al., 2012). However, how rSS affects the motor system remains largely speculative. It is generally believed that the transfer of beneficial effects to sensorimotor behavior elicited by sensory stimulation is based on interconnections between somatosensory and motor cortex (Jones et al., 1978; Stepniewska et al., 1993; Ridding and Uy, 2003; Wu and Kaas, 2003). These interconnections are assumed to elicit a cortical reorganization in the primary motor cortex after stimulation, resulting in increased excitability of the motor cortical representations (Chen et al., 1999; Ridding et al., 2001), in intracortical facilitation (Kobayashi et al., 2003), and in a decrease in intracortical inhibition (Classen et al., 2000), probably modulated by GABAergic neurotransmission (Kaelin-Lang et al., 2002). Since the hand and finger representations of the primary somatosensory cortex have been shown to be directly affected by rSS applied to the fingers (Pleger et al., 2001; Dinse et al., 2003; Kalisch et al., 2008a; Lenz et al., 2012), it is believed that the particular stimulation protocol used induces plastic reorganization in these areas, for a detailed discussion (see Dinse et al., 2011). A signature of such a reorganization also in patients might be the partial restoration of cortical responsiveness found in patient CC, where almost normal tactile-evoked SEPs after 22 weeks of rSS applied to the N. ulnaris innervated finger d5 (little finger) could be recorded. It remains to be seen whether after even longer stimulation times also SEPs could be recorded at d2 (index finger).

An issue of substantial relevance is the question in how far improvements that can be measured in a laboratory surrounding are transferable into everyday life - the major challenge of all intervention strategies. BB reported subjectively improved sensations when touching object surfaces with the fingers, which had not been possible before the intervention. CC, whose incident dated back more than 10 years, showed improvements in all investigated aspects of sensorimotor performance, but reported little impact on changes of self-assessed everyday life activity of the affected extremity. This appears surprising as CC, who was right-hander before the incident, and had shifted to complete left-handedness, showed a clear restoration to mild right-handedness after the intervention. The main reason for this discrepancy might be simple fact that she got so used to not involving the affected hand and arm that she literally forgot to use it, a phenomenon captured in the concept of “compensatory learned non-use of the affected limb” (Grotta et al., 2004; Krakauer, 2006). As a consequence, the individual might not recognize gains of sensorimotor performance induced by an intervention although they can be demonstrated under standardized conditions. Given that, further investigations are necessary to develop additional therapeutic programs to facilitate re-using affected limbs.

Combined, our single case studies based on three individual patients showed that rSS approaches can be applied at the homes of the individuals to provide long-term effective treatment of sensorimotor impairments following cortical lesion. The particular advantage of rSS which does not require active involvement of the participant make this approach a potential candidate even in patients, where the incident dates back many years, and where treatment is required to be applied at a regular schedule of many months.

### Conflict of interest statement

The authors declare that the research was conducted in the absence of any commercial or financial relationships that could be construed as a potential conflict of interest.

## References

[B1] Alegria J. Bertelson P. (1970). Time uncertainty, number of alternatives and particular signal-response pair as determinantof choice reaction time. Acta Psychol. 33, 36–44

[B2] Biernaskie J. Szymanska A. Windle V. Corbett D. (2005). Bi-hemispheric contribution to functional motor recovery of the affected forelimb following focal ischemic brain injury in rats. Eur. J. Neurosci. 21, 989–999 10.1111/j.1460-9568.2005.03899.x 15787705

[B3] Brown C. E. Aminoltejari K. Erb H. Winship I. R. Murphy T. H. (2009). *In vivo* voltage-sensitive dye imaging in adult mice reveals that somatosensory maps lost to stroke are replaced over weeks by new structural and functional circuits with prolonged modes of activation within both the peri-infarct zone and distant sites. J. Neurosci. 29, 1719–1734 10.1523/JNEUROSCI.4249-08.2009 19211879 PMC6666293

[B4] Candia V. Wienbruch C. Elbert T. Rockstroh B. Ray W. (2003). Effective behavioral treatment of focal hand dystonia in musicians alters somatosensory cortical organization. Proc. Natl. Acad. Sci. U.S.A. 100, 7942–7946 10.1073/pnas.1231193100 12771383 PMC164692

[B5] Celnik P. Hummel F. Harris-Love M. Wolk R. Cohen L. G. (2007). Somatosensory stimulation enhances the effects of training functional hand tasks in patients with chronic stroke. Arch. Phys. Med. Rehabil. 88, 1369–1376 10.1016/j.apmr.2007.08.001 17964875

[B6] Chen R. Cohen L. G. Hallett M. (1997). Role of the ipsilateral motor cortex in voluntary movement. Can. J. Neurol. Sci. 24, 284–291 9398974 10.1017/s0317167100032947

[B7] Chen R. Corwell B. Hallett M. (1999). Modulation of motor cortex excitability by median nerve and digit stimulation. Exp. Brain Res. 129, 77–86 10.1007/s002210050938 10550505

[B8] Classen J. Steinfelder B. Liepert J. Stefan K. Celnik P. Cohen L. G. Hess A. Kunesch E. Chen R. Benecke R. Hallett M. (2000). Cutaneomotor integration in humans is somatotopically organized at various levels of the nervous system and is task dependent. Exp. Brain Res. 130, 48–59 10.1007/s002210050005 10638440

[B9] Conforto A. B. Cohen L. G. dos Santos R. L. Scaff M. Marie S. K. (2007). Effects of somatosensory stimulation on motor function in chronic cortico-subcortical strokes. J. Neurol. 254, 333–339 10.1007/s00415-006-0364-z 17345047

[B10] Conforto A. B. Kaelin-Lang A. Cohen L. G. (2002). Increase in hand muscle strength of stroke patients after somatosensory stimulation. Ann. Neurol. 51, 122–125 11782992 10.1002/ana.10070

[B11] Dinse H. R. (2006). Cortical reorganization in the aging brain. Prog. Brain Res. 157, 57–80 17167904 10.1016/s0079-6123(06)57005-0

[B12] Dinse H. R. Boland J. Kalisch T. Kraemer M. Freund E. Beeser E. Hömberg V. Stephan K. M. (2008). Repetitive sensory stimulation training in stroke. Eur. J. Neurol. 15(Suppl. 3), 400

[B13] Dinse H. R. Kalisch T. Ragert P. Pleger B. Schwenkreis P. Tegenthoff M. (2005). Improving human haptic performance in normal and impaired human populations through unattended activation-based learning. ACM Trans. Appl. Percept. 2, 71–88

[B14] Dinse H. R. Kattenstroth J. C. Gattica Tossi M. A. Tegenthoff M. Kalisch T. (2011). Sensory stimulation for augmenting perception, sensorimotor behavior and cognition, in Augmenting Cognition eds Segev I. Markram H. (Lausanne: EPFL Press), 11–39

[B15] Dinse H. R. Kleibel N. Kalisch T. Ragert P. Wilimzig C. Tegenthoff M. (2006). Tactile coactivation resets age-related decline of human tactile discrimination. Ann. Neurol. 60, 88–94 10.1002/ana.20862 16685697

[B16] Dinse H. R. Ragert P. Pleger B. Schwenkreis P. Tegenthoff M. (2003). Pharmacological modulation of perceptual learning and associated cortical reorganization. Science 301, 91–94 10.1126/science.1085423 12843392

[B17] Dobkin B. H. (2004). Strategies for stroke rehabilitation. Lancet Neurol. 3, 528–536 10.1016/S1474-4422(04)00851-8 15324721 PMC4164204

[B18] Ebied A. M. (2003). The role of cutaneous sensation in the motor function of the hand. J. Orthop. Res. 22, 862–866 10.1016/j.orthres.2003.12.005 15183446

[B19] Elbert T. Pantev C. Wienbruch C. Rockstroh B. Taub E. (1995). Increased cortical representation of the fingers of the left hand in string players. Science 270, 305–307 10.1126/science.270.5234.305 7569982

[B20] Folstein M. F. Folstein S. E. McHugh P. R. (1975). “Mini-mental state”. A practical method for grading the cognitive state of patients for the clinician. J. Psychiatr. Res. 12, 189–198 10.1016/0022-3956(75)90026-6 1202204

[B21] Fraser C. Power M. Hamdy S. Rothwell J. Hobday D. Hollander I. Tyrell P. Hobson A. Williams S. Thompson D. (2002). Driving plasticity in human adult motor cortex is associated with improved motor function after brain injury. Neuron 34, 831–840 10.1016/S0896-6273(02)00705-5 12062028

[B22] Gerloff C. Cohen L. G. Floeter M. K. Chen R. Corwell B. Hallett M. (1998). Inhibitory influence of the ipsilateral motor cortex on responses to stimulation of the human cortex and pyramidal tract. J. Physiol. 510(Pt 1), 249–259 10.1111/j.1469-7793.1998.249bz.x 9625881 PMC2231019

[B23] Grotta J. C. Noser E. A. Ro T. Boake C. Levin H. Aronowski J. Schallert T. (2004). Constraint-induced movement therapy. Stroke 35, 2699–2701 10.1161/01.STR.0000143320.64953.c4 15375308

[B24] Hackel M. E. Wolfe G. A. Bang S. M. Canfield J. S. (1992). Changes in hand function in the aging adult as determined by the Jebsen Test of Hand Function. Phys. Ther. 72, 373–377 1631206 10.1093/ptj/72.5.373

[B25] Hamdy S. Rothwell J. C. Aziz Q. Singh K. D. Thompson D. G. (1998). Long-term reorganization of human motor cortex driven by short-term sensory stimulation. Nat. Neurosci. 1, 64–68 10.1038/264 10195111

[B26] Hashimoto I. Suzuki A. Kimura T. Iguchi Y. Tanosaki M. Takino R. Haruta Y. Taira M. (2004). Is there training-dependent reorganization of digit representations in area 3b of string players? Clin. Neurophysiol. 115, 435–447 10.1016/S1388-2457(03)00340-7 14744586

[B27] Jäncke L. Schlaug G. Steinmetz H. (1997). Hand skill asymmetry in professional musicians. Brain Cogn. 34, 424–432 10.1006/brcg.1997.0922 9292190

[B28] Jäncke L. Shah N. J. Peters M. (2000). Cortical activations in primary and secondary motor areas for complex bimanual movements in professional pianists. Brain Res. Cogn. Brain Res. 10, 177–183 10.1016/S0926-6410(00)00028-8 10978706

[B29] Jebsen R. H. Taylor N. Trieschmann R. B. Trotter M. J. Howard L. A. (1969). An objective and standardized test of hand function. Arch. Phys. Med. Rehabil. 50, 311–319 5788487

[B30] Johansson B. B. (2011). Current trends in stroke rehabilitation. A review with focus on brain plasticity. Acta Neurol. Scand. 123, 147–159 10.1111/j.1600-0404.2010.01417.x 20726844

[B31] Jones E. G. Coulter J. D. Hendry S. H. (1978). Intracortical connectivity of architectonic fields in the somatic sensory, motor and parietal cortex of monkeys. J. Comp. Neurol. 181, 291–347 10.1002/cne.901810206 99458

[B32] Kaelin-Lang A. Luft A. R. Sawaki L. Burstein A. H. Sohn Y. H. Cohen L. G. (2002). Modulation of human corticomotor excitability by somatosensory input. J. Physiol. 540, 623–633 10.1113/jphysiol.2001.012801 11956348 PMC2290238

[B33] Kalisch T. Kattenstroth J. C. Kowalewski R. Tegenthoff M. Dinse H. R. (2011a). Cognitive and tactile factors affecting human haptic performance in later life. PLoS ONE 7:e30420 10.1371/journal.pone.0030420 22291952 PMC3264587

[B34] Kalisch T. Richter R. Lenz M. Kattenstroth J. C. Kolankowska I. Tegenthoff M. Dinse H. R. (2011b). Questionnaire-based evaluation of everyday competence in older adults. Clin. Interv. Aging 6, 37–46 10.2147/CIA.S15433 21472090 PMC3066251

[B35] Kalisch T. Tegenthoff M. Dinse H. (2007). Differential effects of synchronous and asynchronous multifinger coactivation on human tactile performance. BMC Neurosci. 8, 58 10.1186/1471-2202-8-58 17663778 PMC1949832

[B36] Kalisch T. Tegenthoff M. Dinse H. R. (2008a). Improvement of sensorimotor functions in old age by passive sensory stimulation. Clin. Interv. Aging 3, 673–690 10.2147/CIA.S3174 19281060 PMC2682400

[B37] Kalisch T. Ragert P. Schwenkreis P. Dinse H. R. Tegenthoff M. (2008b). Impaired tactile acuity in old age is accompanied by enlarged hand representations in somatosensory cortex. Cereb. Cortex 19, 1530–1538 10.1093/cercor/bhn190 19008462

[B38] Kalisch T. Tegenthoff M. Dinse H. R. (2010). Repetitive electric stimulation elicits enduring improvement of sensorimotor performance in seniors. Neural Plas. 2010, 11 10.1155/2010/690531 20414332 PMC2855030

[B39] Kalisch T. Wilimzig C. Kleibel N. Tegenthoff M. Dinse H. R. (2006). Age-related attenuation of dominant hand superiority. PLoS ONE 1:e90 10.1371/journal.pone.0000090 17183722 PMC1762407

[B40] Kobayashi M. Ng J. Théoret H. Pascual-Leone A. (2003). Modulation of intracortical neuronal circuits in human hand motor area by digit stimulation. Exp. Brain Res. 149, 1–8 10.1007/s00221-002-1329-9 12592498

[B41] Kowalewski R. Kattenstroth J. C. Kalisch T. Dinse H. R. (2012). Improved acuity and dexterity but unchanged touch and pain thresholds following repetitive sensory stimulation of the fingers. Neural Plast. 2012, 974504 10.1155/2012/974504 22315693 PMC3270448

[B42] Krakauer J. W. (2006). Motor learning: its relevance to stroke recovery and neurorehabilitation. Curr. Opin. Neurol. 19, 84–90 16415682 10.1097/01.wco.0000200544.29915.cc

[B43] Lederman S. J. Klatzky R. L. (2004). Haptic identification of common objects: effects of constraining the manual exploration process. Percept. Psychophys. 66, 618–628 15311661 10.3758/bf03194906

[B44] Lenz M. Tegenthoff M. Kohlhaas K. Stude P. Höffken O. Tossi M. Kalisch T. Dinse H. (2012). Increased excitability of somatosensory cortex in aged humans is associated with impaired tactile acuity. J. Neurosci. 32, 1811–1816 10.1523/JNEUROSCI.2722-11.2012 22302820 PMC6703354

[B45] Liepert J. Hamzei F. Weiller C. (2000). Motor cortex disinhibition of the unaffected hemisphere after acute stroke. Muscle Nerve 23, 1761–1763 10.1002/1097-4598(200011)23:11<1761::AID-MUS14>3.0.CO;2-M 11054757

[B46] Liepert J. Uhde I. Gräf S. Leidner O. Weiller C. (2001). Motor cortex plasticity during forced-use therapy in stroke patients: a preliminary study. J. Neurol. 248, 315–321 10.1007/s004150170207 11374097

[B47] Lincoln N. Leadbitter D. (1979). Assessment of motor function in stroke patients. Physiotherapy 65, 48–51 441189

[B48] McDonnell M. N. Ridding M. C. (2006). Afferent stimulation facilitates performance on a novel motor task. Exp. Brain Res. 170, 109–115 10.1007/s00221-005-0192-x 16328288

[B49] Montoya P. Sitges C. (2006). Affective modulation of somatosensory-evoked potentials elicited by tactile stimulation. Brain Res. 1068, 205–212 10.1016/j.brainres.2005.11.019 16364261

[B49a] Murphy T. H. Corbett D. (2009). Plasticity during stroke recovery: from synapse to behaviour. Nat. Rev. Neurosci. 10, 861–872 10.1038/nrn2735 19888284

[B50] Nudo R. J. Wise B. M. SiFuentes F. Milliken G. W. (1996). Neural substrates for the effects of rehabilitative training on motor recovery after ischemic infarct. Science 272, 1791–1794 10.1126/science.272.5269.1791 8650578

[B51] Pavlides C. Miyashita E. Asanuma H. (1993). Projection from the sensory to the motor cortex is important in learning motor skills in the monkey. J. Neurophysiol. 70, 733–741 8410169 10.1152/jn.1993.70.2.733

[B52] Pleger B. Dinse H. R. Ragert P. Schwenkreis P. Malin J. P. Tegenthoff M. (2001). Shifts in cortical representations predict human discrimination improvement. Proc. Natl. Acad. Sci. U.S.A. 98, 12255–12260 10.1073/pnas.191176298 11593042 PMC59801

[B53] Pleger B. Foerster A. F. Ragert P. Dinse H. R. Schwenkreis P. Malin J. P. Nicolas V. Tegenthoff M. (2003). Functional imaging of perceptual learning in human primary and secondary somatosensory cortex. Neuron 40, 643–653 10.1016/S0896-6273(03)00677-9 14642286

[B54] Ragert P. Franzkowiak S. Schwenkreis P. Tegenthoff M. Dinse H. R. (2008). Improvement of tactile perception and enhancement of cortical excitability through intermittent theta burst rTMS over human primary somatosensory cortex. Exp. Brain Res. 184, 1–11 10.1007/s00221-007-1073-2 17680239

[B55] Raven J. C. (1938). Progressive Matrices. London: Lewis and Co

[B56] Ridding M. C. Uy J. (2003). Changes in motor cortical excitability induced by paired associative stimulation. Clin. Neurophysiol. 114, 1437–1444 10.1016/S1388-2457(03)00115-9 12888026

[B57] Ridding M. C. Brouwer B. Miles T. S. Pitcher J. B. Thompson P. D. (2000). Changes in muscle responses to stimulation of the motor cortex induced by peripheral nerve stimulation in human subjects. Exp. Brain Res. 131, 135–143 10.1007/s002219900269 10759179

[B58] Ridding M. C. McKay D. R. Thompson P. D. Miles T. S. (2001). Changes in corticomotor representations induced by prolonged peripheral nerve stimulation in humans. Clin. Neurophysiol. 112, 1461–1469 10.1016/S1388-2457(01)00592-2 11459686

[B59] Smith P. Dinse H. Kalisch T. Johnson M. Walker-Batson D. (2009). Effects of repetitive electrical stimulation to treat sensory loss in persons poststroke. Arch. Phys. Med. Rehabil. 90, 2108–2111 10.1016/j.apmr.2009.07.017 19969176

[B60] Steingrueber H. J. (1971). Zur messung der haendigkeit. Zeitschrift fuer Experimentelle und Angewandte Psychologie 18, 337–357 5561973

[B61] Stepniewska I. Preuss T. M. Kaas J. H. (1993). Architectonics, somatotopic organization, and ipsilateral cortical connections of the primary motor area (M1) of owl monkeys. J. Comp. Neurol. 330, 238–271 10.1002/cne.903300207 7684050

[B62] Taub E. Uswatte G. Elbert T. (2002). New treatments in neurorehabilitation founded on basic research. Nat. Rev. Neurosci. 3, 228–236 10.1038/nrn754 11994754

[B63] Taub E. Uswatte G. Pidikiti R. (1999). Constraint-induced movement therapy: a new family of techniques with broad application to physical rehabilitation–a clinical review. J. Rehabil. Res. Dev. 36, 237–251 10659807

[B64] Webster B. R. Celnik P. A. Cohen L. G. (2006). Noninvasive brain stimulation in stroke rehabilitation. NeuroRx 3, 474–481 10.1016/j.nurx.2006.07.008 17012061 PMC3593409

[B65] Wienbruch C. Candia V. Svensson J. Kleiser R. Kollias S. S. (2006). A portable and low-cost fMRI compatible pneumatic system for the investigation of the somatosensensory system in clinical and research environments. Neurosci. Lett. 398, 183–188 10.1016/j.neulet.2006.01.025 16469438

[B66] Wilimzig C. Ragert P. Dinse H. R. (2012). Cortical topography of intracortical inhibition influences the speed of decision making. Proc. Natl. Acad. Sci. U.S.A. 109, 3107–3112 10.1073/pnas.1114250109 22315409 PMC3286921

[B67] Woldag H. Waldmann G. Heuschkel G. Hummelsheim H. (2003). Is the repetitive training of complex hand and arm movements beneficial for motor recovery in stroke patients? Clin. Rehabil. 17, 723–730 10.1191/0269215503cr669oa 14606737

[B68] Wu C. W. Kaas J. H. (2003). Somatosensory cortex of prosimian Galagos: physiological recording, cytoarchitecture, and corticocortical connections of anterior parietal cortex and cortex of the lateral sulcus. J. Comp. Neurol. 457, 263–292 10.1002/cne.10542 12541310

[B69] Wu C. W. Seo H. J. Cohen L. G. (2006). Influence of electric somatosensory stimulation on paretic-hand function in chronic stroke. Arch. Phys. Med. Rehabil. 87, 351–357 10.1016/j.apmr.2005.11.019 16500168

[B70] Zhan S. Ottenbacher K. J. (2001). Single subject research designs for disability research. Disabil. Rehabil. 23, 1–8 11213316 10.1080/09638280150211202

